# Preschool Children’s Dietary Patterns Are Associated with Food Shopping Establishments: The SENDO Project

**DOI:** 10.3390/foods13182930

**Published:** 2024-09-16

**Authors:** Marina Ródenas-Munar, Silvia García, Violeta Cepeda, Nerea Martín-Calvo, Josep A. Tur, Cristina Bouzas

**Affiliations:** 1 Research Group on Community Nutrition and Oxidative Stress, University of the Balearic Islands-IUNICS, 07122 Palma de Mallorca, Spain; m.rodenas@uib.es (M.R.-M.);; 2 Health Research Institute of the Balearic Islands (IdISBa), 07120 Palma de Mallorca, Spain; 3 CIBER Fisiopatología de la Obesidad y Nutrición (CIBEROBN), Instituto de Salud Carlos III (ISCIII), 28029 Madrid, Spain; 4 Department of Preventive Medicine and Public Health, University of Navarra, Instituto de Investigación Sanitaria de Navarra (IdiSNA), 31008 Pamplona, Spain; 5 Center for Nutrition Research, University of Navarra, 31008 Pamplona, Spain

**Keywords:** hypermarket, supermarket, specialised establishment, dietary habits, foods, children

## Abstract

***Background:*** Eating habits established during the preschool stage may last a lifetime, underlining the importance of choosing and purchasing healthy foods for proper nutrition. ***Aim:*** To assess the associations between place of food purchase and dietary intake and quality in Spanish preschool children. ***Methods:*** A cross-sectional analysis was carried out within the framework of the SEguimiento del Niño para un Desarrollo Óptimo (SENDO) project. Data were collected using (1) the food frequency questionnaire (FFQ), from which dietary nutrient intake and diet quality (NOVA System classification and KIDMED index) were analysed, and (2) the baseline food habits questionnaire, which asked about the food purchase outlet. Data were analysed by logistic regression, considering the intra-group correlation between siblings and using the type of food purchase outlet as a grouping variable. ***Results:*** Children from families shopping in hypermarkets consumed fewer nutrients, less minimally processed foods, as well as less fruit and vegetables than children from families shopping in specialised shops. ***Conclusions:*** Food shopping in specialised outlets can improve the quality of children’s diets, promoting healthier habits such as greater adherence to the Mediterranean Diet and increasing the consumption of minimally processed foods. This has a positive impact on children’s long-term health.

## 1. Introduction

In the 21st century context, the situation of child undernutrition has been analysed [1]. Globally, one third of <5-year-old children younger is undernourished or overweight, while around half of them suffers from hidden hunger (micronutrient deficiencies). Nearly 50 million children are acutely undernourished; moreover, hundreds of millions of children and women suffer from micronutrient deficiencies (hidden hunger), while obesity rates are rising rapidly. This situation of child undernutrition has a negative impact on children’s ability to achieve adequate growth and development and has become a main risk factor of diseases worldwide. Overcoming this challenge would require addressing undernutrition at all stages of a child’s life and prioritising their specific needs in food systems, as well as in health, water and sanitation, education and social protection support systems. In this regard, some of these targets are intended to be achieved in the 2030 Agenda of the Sustainable Development Goals [2]. Improving child nutrition therefore implies that food systems provide nutritious, safe, accessible, and sustainable food for all children [1]. In parallel to these challenges, the world is facing a pandemic of childhood obesity that is growing at an alarming rate. In recent decades, obesity rates in children and adolescents have risen sharply, putting the health of new generations at risk [3,4]. This increase is largely due to an increase in the consumption of ultra-processed foods and a more sedentary lifestyle. However, scientific evidence shows that there are multiple factors that favour the development of childhood obesity [5]. This situation, which coexists with undernutrition and malnutrition, represents one of the greatest challenges for global public health [3].

Childhood food and nutrition are crucial for children’s growth, cognitive development, academic performance, and future productivity. The preschool age is a crucial period, as they are growing, for establishing and instilling eating habits, preferences and behaviours that can have a final impact on health children life. In this regard, parents play an important role in promoting the health of their preschool children and are responsible for encouraging appropriate eating behaviours [6,7,8,9]. Even so, most children and adolescents continue to have diets that lack the necessary nutrients. This has important implications for metabolic diseases, overweight, obesity, and other diet-related conditions [10,11]. One article found that excessive consumption of ultra-processed foods (UPF) was associated with an increased likelihood of inadequate intake of at least 3 micronutrients during childhood [12]. Findings from another study indicate links between the consumption of UPF and negative health effects in children and adolescents [13]. In addition, another study indicated that poor adherence to the Mediterranean Diet (Mediet) increases the likelihood of hypertriglyceridaemia, central obesity and insulin resistance in a Greek paediatric population [14].

In this context, the environments in which we are living influence how and what we eat, as they affect our eating behaviour through a variety of factors, such as food availability and accessibility, food advertising and marketing, prices, culture, food education, and policies and regulations, all of which can shape food preferences and eating habits, both in terms of nutritional quality and consumption patterns. All these factors can shape food preferences and eating habits, both in terms of nutritional quality and consumption patterns [10,15,16]. Thus, the place where food is purchased is another factor that can influence eating behaviour. Shopping in department stores (HPM and SPM) has its advantages and disadvantages. The main attraction of shopping at these sites is the convenience of having all products in one place, such as food, cleaning products, electronics, clothing, etc. In addition, the economy of scale of these establishments means that prices are lower. The main disadvantages of shopping in these establishments are the low quality and lack of specialisation of food products. On the other hand, in specialised establishments (SES) the main advantages are the quality and freshness of the products, as well as offering local and artisanal products that can only be found in these establishments, and they also have the capacity to offer a more personalised service to the consumer. But the main disadvantages are limited variety and higher prices [17,18]. The choice of where to shop can have an impact on people’s health. Shopping in an HPM and/or SPM would be a poorer food choice due to the wide range of UPF products and products of low nutritional quality. These processed and UPF products are composed of unhealthy ingredients and are high in fat, sugars, sodium, etc., thus posing a long-term health risk [19,20]. On the other hand, shopping in specialised establishments means buying fresh, quality products with higher nutritional value, which can contribute to a healthier and more balanced diet. Therefore, the choice of where to shop affects not only convenience and cost, but also the health of the consumer [17,18]. In the current study, we referred to an HPM that offers a wide range of food and non-food products, with large surfaces and a diversity of sections. The SPM is a smaller retail outlet than an HPM, offering a variety of commodities and groceries, usually in a more limited space. And SES (in markets, neighbourhood shops, etc.) for the sale of specific products of a food category, such as butchers, fishmongers or greengrocers, among others. In recent years, the trend among Spanish consumers has been to opt less and less for traditional markets and local shops, preferring instead to buy fresh products such as meat, fish or fruit in SPMs, and to complete their purchases in larger HPMs [17].

There are lots of factors influencing consumers’ perception of food safety, such as sex, age, employment, living site, and education level, as well as social and information media, expert consultations, and official websites, among others. Consumers’ perception is largely affected by the availability of information, and they may be more inclined to choose trustworthy food brands or products [21].

If the healthy food environment at home can influence children’s eating habits, the place of shopping is part of the availability and access to food that the child may have at home, therefore, it is important to see where families shop and how it can influence the child’s dietary intake and nutritional quality.

Therefore, the current study aimed to analyse the association between the place of food shopping and dietary quality in Spanish preschool children (4 years old). That is, whether parents’ place of food shopping (HPM, SPM, SES) was associated with a more or less healthy or nutritious diet in pre-school children in Spain.

## 2. Materials and Methods

### 2.1. Study Design

A cross-sectoral analysis was carried out in the framework of the project “SEguimiento del Niño para un Desarrollo Óptimo” (SENDO), a national, versatile, prospective and dynamic research. It focuses on the study of the effects of eating habits and lifestyle on the health of children and adolescents. More information on the SENDO study can be found at http://www.proyectosendo.es (accessed date: 5 September 2024).

### 2.2. Participants, Recruitment, Randomization and Ethics

Participants at the beginning of the study were children aged 4–5 years living in Spain. These were the two inclusion criteria, and the only exclusion criterion was the lack of access to a device connected to the Internet to complete the questionnaires. Participants were invited to enter the cohort by their paediatrician in primary care health centres or by the research team in early childhood education centres. Recruitment began in 2015 and is ongoing. For the current study, data from participants recruited between January 2015 and June 2023, from the baseline questionnaire (Q0) was used. A total sample of 1208 participants was obtained, who were recruited either through their paediatricians in the health centres or by the research groups through the schools. Missing data on dietary habits (*n* = 60) were excluded as participants did not answer or did not complete the questionnaire. This resulted in a final sample of 1148 participants. Baseline information is collected through an extensive questionnaire (Q0) covering various aspects such as personal and family history, socio-demographic background, physical characteristics, eating habits, behaviours, lifestyle, physical activity and personality traits. Parents/legal guardians of participants complete this questionnaire online and enter it into a secure database on the SENDO website. Each year, parents were asked to complete a new online questionnaire, which changes depending on the age of the child until he/she reaches the age of majority.

The SENDO study followed the Declaration of Helsinki and was approved by the Clinical Research Ethics Committee of Navarra (Pyto 2016/122; 2 February 2017). Parents of participants signed an informed consent form before entering the SENDO project. The information managed by the researchers will be contained in a database coded, in which the identification of the participants will not be possible. Only the principal investigator knew the personal data of the participants, which was responsible for its custody. This information was kept under lock and key in a secure location designated for this purpose. The processing, communication and transfer of personal data of all participating subjects complied with the provisions of the Regulation (EU) 2016/679 of the European Parliament and of the Council of 27 April 2016 on the protection of natural persons with regard to the processing of personal data and on the free movement of such data, and repealing Directive 95/46/EC (General Data Protection Regulation). According to the current legislation, participants could exercise the rights of access, rectification, cancellation, and opposition of data (ARCO standards) by contacting the SENDO research team through the telephone numbers and email that participants could find at the end of informed signed consent.

### 2.3. Food Purchasing Establishment and Habits

The baseline questionnaire consists of five parts, one of which concerns questions on eating habits. This section asked about the type of establishment where people shopped (“In general, in which type of establishment do you shop?”). From the three possible answers: hypermarket, supermarket and specialised outlets, the grouping variable for this study was obtained.

Information was also collected on the factors that most influence the choice of food shop (Distance, Prices, Quality of products, Variety of the offer, Other) and the factors that most influenced the choice of food when shopping (Organic, Sustainable, Brand, Price, Nutritional composition, Product presentation, Other).

### 2.4. Dietary Assessment

Dietary information was collected using a previously validated semi-quantitative Food Frequency Questionnaire (FFQ), specifically designed for the preschool population [22], which included 149 foods, organised by 10 food groups (dairy, eggs, meat and fish, vegetables, fruit, pulses and cereals, oils and fats, sweets and snacks, beverages, pastries and cakes, miscellaneous). For each food, the serving size was specified. Parents reported the frequency with which their children had consumed each food during the previous year by selecting one of nine consumption frequencies, ranging from “never or hardly ever” to “6 or more times a day”.

A team of specialised dieticians calculated the nutritional content of each food to obtain the intake of each nutrient. This calculation consisted of multiplying the frequency of consumption by the edible portion and the nutritional composition of the specified portion size. For these calculations, data from updated food composition tables in Spain [23] and online databases [24,25] were used to ensure accuracy and relevance.

The amount consumed of each food (g/day) for dietary intake was also obtained. This was calculated by multiplying the frequency of consumption (first converted to daily frequency of consumption) by the edible portion of each of the foods and then adding the foods from each of the food groups to obtain the total g/day.

Amount consumed (g/day) = Daily frequency of consumption × Grams of edible portion size

Total g/day of the food group = amount consumed (g/day of food 1) + amount consumed (g/day of food 2) + amount consumed (g/day of food 3)

### 2.5. Diet Quality

Information on diet quality was obtained using the NOVA System classification [26] and the KIDMED index [27]. All FFQ foods were classified according to their degree of processing using the NOVA System, which consists of 4 groups (see Table S1). The first NOVA group includes unprocessed or natural foods and minimally processed foods; the second NOVA group includes processed culinary ingredients (oils, fats, salt and sugar). The third NOVA group includes processed foods (incorporating/adding ingredients from group 2) and the fourth NOVA group includes UPF and beverage products (industrial formulations composed of substances extracted or derived from food, or substances synthesised in laboratories). The KIDMED index was obtained to estimate participants’ adherence to the MedDiet and to assess dietary habits, following the main principles of this dietary pattern. The score was obtained from dichotomous responses (Yes/No) to 16 items. Of these items, four (Q1, Q4, Q11, Q14) had a negative connotation when answered affirmatively (−1 point), the remaining items were scored with one point when answered affirmatively. This score was classified as high adherence to MedDiet (8 or more points), medium (between 4 and 7 points) or low adherence (3 or less points). The administered questionnaires can be provided upon demand to the researcher N.M.-C. (nmartincalvo@unav.es).

### 2.6. Statistics

The statistical package Statistical Package for the Social Sciences version 29.0 (IBM SPSS Statistics for Windows, Armonk, NY, USA: IBM Corp) was used to perform the statistical analyses. A total of 1148 subjects were analysed. Participants were classified into three groups according to the type of establishment where they bought food (HPM, SPM and SES). Analyses were conducted considering the intra-group correlation between siblings.

Quantitative variables were expressed as mean and standard deviation (SD) and differences between groups were analysed by ANOVA and Bonferroni post-hoc. Between-group mean differences were adjusted for mother’s age and father’s educational level using the Unianova test as potential confounders. Analyses were performed after a logarithmic transformation of the data, although the article shows the data without transformation for ease of understanding.

Qualitative variables were expressed as sample frequencies and percentages, and differences were calculated using the chi-square test (χ^2^). Logistic regression analysis with odds ratio (OR) estimation and 95% confidence interval (CI) was calculated to assess the association between food shopping outlet and diet quality (KIDMED questionnaire and its score); and to assess the association between food shopping outlet and factors influencing outlet and food choice.

Potential confounding factors have been considered in Table 1 (socio-demographic factors). Subsequently, these confounding factors that may have an effect (such as the child’s and mother’s age and the father’s level of education) were considered in the rest of the analysis. Quantitative variables were converted into dichotomous variables using the 50th percentile and then analysed with the OR. The OR was calculated twice: first unadjusted (crude OR) and second adjusted for child’s and mother’s age and father’s educational level (adjusted OR) as potential confounders. All results were considered significant if the *p*-value was <0.05.

## 3. Results

Table 1 shows the socio-demographic characteristics of the participants and their parents according to the grocery shop. The mother’s average age and the child’s average age were both higher in the group of those who shopped at SES. The average educational level of the parents was higher in those who shopped at SES.

Table 2 shows the factors influencing the choice of outlet and food shopping according to the food shopping outlet. The products’ quality was the most influential factor in all groups to choose the outlet. Product variety was the second most influential factor in the choice of outlet for those who shopped at HPM and SES. Nutritional composition was the most influential factor in food choice (highest percentage) in all groups. Brand and price were the second most influential factors in food choice for HPM and SPM group. Factors that also influenced food choice were organic products and price in the SES group.

Table 3 shows the children’s dietary intake according to the food purchase establishment. The mean amount (g/day) of vegetable, olive oil and fruit consumption were significantly higher in the SES group. The mean consumption (g/day) of processed meat was significantly higher in the HPM group. Higher consumption (mean amount in g/day) of sweets and cakes, and prepared food was observed in the HPM and SPM groups.

Table 4 shows the NOVA System diet quality (g/day) of the participants according to the food purchasing establishment. In the SES group, a significantly higher consumption (mean amount in g/day) of unprocessed or minimally processed food (NOVA Group 1) was observed.

Table 5 shows the nutrient intake (daily amount) of the children according to the food purchase outlet. In the HPM group the mean daily intake was significantly higher in saturated fatty acids. The mean intake (daily amount) of nutrients was significantly higher in the SES group for folate, fibre, vitamins (B_6_, C and E), magnesium, potassium, chromium.

Table 6 shows the association between dietary habits and place of purchase. The HPM and SPM groups were less likely to consume cereal at breakfast, eat a second fruit and eat vegetables one or more times a day than the SES group, with significant effects (protective factors: 95% Confidence Interval < 1). Those in the SPM group were less likely to consume fish regularly than those in the SES group. The SPM group was more likely to go to fast food restaurants (more than once a week) than the SES group. Adjusting for confounding factors, the SPM group was less likely to eat pastries for breakfast than the SES group. The SPM group was positively associated with lower MedDiet adherence than the SES group. The HPM and SPM groups were negatively associated with high MedDiet adherence relative to the SES group.

Table 7 shows the association between the factors influencing the choice of outlet and food and the shopping outlet. The HPM and SPM groups were more likely to choose distance, price. and product variety as influential factors in choosing where to buy food than those in the SES group. The HPM and SPM groups were less likely to choose product quality as an influential factor in deciding where to buy food than those in the SES group. Those in HPM and SPM groups were negatively associated with the ecological factor in choosing food. HPM was also negatively associated with the sustainable factor for food choice. The HPM and SPM groups were positively associated with price as an influencing factor for food choice, as well as with brand as an influencing factor in the HPM group in comparison with the SES group.

## 4. Discussion

The main results of this current study show that children from families shopping at HPM or SPM had a lower daily intake of micronutrients and a lower consumption (g/day) of unprocessed or minimally processed foods. They showed lower adherence to DietMed and lower consumption of fruit and vegetables than children of families who shopped at SES. Families who choose to shop at HPM and SPM tended to look more at price, product variety, and distance when choosing where to shop, as well as brand and price when choosing food to fill the shopping basket, compared to households shopping in SES.

So far, no recent or enough evidence was found to analyse the place of families’ food shopping with dietary intake and nutritional quality (quantitatively) of their pre-school children at national level. However, the scientific evidence so far suggests that families who shop at the SES consumed more unprocessed or minimally processed (fresh) foods and therefore followed healthier dietary patterns that they will be able to pass on to their children. In the same way, and as suggested throughout this study, HPMs and SPMs offer a wide range of processed and UPF products, which are composed of large amounts of fats, sugars, etc. They are considered unhealthy and of low nutritional quality. These are considered unhealthy and of low nutritional quality. Therefore, if families tend to shop in these types of establishments, their shopping cart will be of poorer quality because of the products offered and therefore their diets and food will be less healthy [17,18,19,20,21,22,23,24,25,26,27,28,29,30]. Consequently, consumers’ health will be affected, with negative repercussions on children’s health. Research suggests that unhealthy diets are associated with long-term disease [10,11,28,29].

The current study showed that factors such as product quality and variety influence households’ choice of purchase place, as well as the nutritional composition and price of the food basket, according to the three outlets (HPM, SPM or SES). One study showed similar results, but more generalised [17]. Factors influencing the choice of purchase place were price, availability and variety of food, whereas price, promotions, best-before date, and healthy food were valued at purchase time [17]. In a systematic review, availability, accessibility, and price were also found to be determining factors in the outlet choice. Food quality and shop characteristics also played an important role in purchasing decisions [30]. 

In recent decades, there were significant changes in the food system, influenced by socio-economic and cultural forces. This has led to a gradual shift from traditional diets based on fresh and minimally processed foods to dietary patterns dominated by industrialised products [31]. These changes are also due to the need to optimise time and energy, which is why people choose to buy products in HPMs and SPMs due to the comfort and convenience provided by these establishments [18]. It should also be borne in mind that food production techniques have also evolved, partly affecting the quality of the food, which is more energy-dense, with more salt, fat, etc., and more processed, the more processed the food is. These are more processed foods, which can be found in large supermarkets [32]. The trend in these dietary patterns was also observed in the results of the present study, with more households opting for SPM.

Modern retail outlets (including supermarkets, hypermarkets, convenience stores, and fast-food restaurants) play an important role in increasing the consumption of highly processed and high-calorie foods. These foods have a lower healthy profile than unprocessed foods [33]. Consequently, our current results on food intake showed that a healthier dietary pattern is followed by people who bought groceries mainly in speciality shops, as opposed to shoppers in hypermarkets and supermarkets.

Previous research in developing countries (Guatemala, Kenya, and Zambia) reported that modern SPM are associated with lower intake/consumption of unprocessed foods [33,34,35]. In addition, households that frequent modern retail outlets tend to consume fewer vegetables and pulses, but more meat, dairy, sugar and beverages than those that do not frequent SPM [33]. Households that shopped in modern SPM spent lower proportion of expenditures on unprocessed foods than those who did not shop in modern SPM [33,34,35]. These results are consistent with current findings. Current consumption of unprocessed foods was lower in children whose families shopped in HPM and SPM and, although the foods may differ from those in the current study being in developing countries, they were similar in terms of lower consumption of vegetables and higher consumption of sweets [33,34,35].

Several studies investigated how access, distance, and expansion of HPM and SPM influenced diet quality [33,34,35,36]. These findings suggested that as SPM and HPM expanded and became more accessible to families, families tended to opt for lower healthy diets. Moreover, children were found to be more exposed to unhealthy establishments both at home and school [36]. According to the current results, distance is also a factor influencing the choice of outlet and thus food. Consequently, shopping in SPM and HPM was associated with a lower adherence to a healthy dietary pattern (such as the MedDiet), which is characterised by a higher consumption of plant-based foods (fruits, vegetables, legumes and nuts), olive oil as the main fat, as well as adequate consumption of fish and wholegrain carbohydrates, reducing the consumption of processed products. It was also found that access to specialised health food shops is related to better diet quality and greater adherence to the MedDiet.

However, not all SPM are the same. A UK study found that, compared to households opting for lower-priced SPM, those choosing higher-end SPM bought more energy from fruit and vegetables, and reduced energy from low healthy foods and drinks. Thus, purchasing higher-priced SPM was associated with higher quality diets and purchasing lower-priced SPM was associated with a less nutritious diet, with lower consumption of vegetables and fruit [37]. In another study, similar results were observed in those who purchased from high cost/range SPM had higher quality diets than those who purchased from low cost/range SPM [38].

Previous research showed that one of the influential aspects of the family environment is the parental dietary patterns, which can influence children’s food preferences and choices [39,40,41,42]. One study quantified change in the family food environment and identified a number of factors (nutritional knowledge, perceived responsibility and restraint) that were associated with a decrease in children’s intake of saturated fat [39]. Another study highlights the im-portance of parental nutrition education in improving their own and their children’s dietary attitudes [40]. It was reported that the fruits and vegetables consumption reported by parents was positively correlated with children’s overall Healthy Eating Index (HEI) score, while the number of fizzy drinks and caloric snacks reported by parents was negatively correlated with children’s overall Healthy Eating Index score [41]. It was found that good parental diet quality was associated with higher adherence of children and adolescents to the MedDiet, as well as higher consumption of fruits, vegetables, fish, legumes and nuts [42]. This highlights the importance of purchasing food from specialised outlets, since it is associated with better dietary intake and quality and higher adherence to the MedDiet, as observed in the current findings.

### Strengths and Limitations of the Study

The focus of the current study is highly relevant, since it assessed the place of food shopping, to develop intervention strategies promoting healthy eating and children’s future health. This work contributes significantly to the understanding of how place of purchase food is associated with the 4-year-old children dietary pattern. To our knowledge, this is the first study that quantitatively assesses the dietary intake and nutritional quality of preschool children in Spain and associates it with parents’ place of food purchase. The current study has several limitations. Firstly, as it is a cross-sectional design, it is not possible to provide evidence of causality. Secondly, the questionnaires were self-administered, which introduces possible biases and measurement errors. Thirdly, the current sample may not be fully representative of the Spanish population. However, it is important to bear in mind that cohort studies do not necessarily demand representative samples of the population studied. As long as the variability of the sample allows comparison of the hypotheses under investigation, representativeness is not essential [43]. Fourthly, the FFQs, being self-completed, could lead to inaccuracies in the measurements. However, they were validated [22] and used in previous epidemiological research and are therefore considered the most practical and feasible instruments for assessing habitual eating patterns. Finally, the FFQ does not collect information on food processing, so some misclassification cannot be ruled out. However, to reduce the possibility of misclassification errors, two different researchers conducted the food classification independently and any disagreements were resolved by consensus.

## 5. Conclusions

The current study assessed associations between food purchasing establishments and the quality of children’s diets; then, it can be concluded that those who shop at HPM and SPM are guided more by price and brand, and those who go to SES by product quality. In addition, children whose families shopped at HPM consumed fewer nutrients, less fruit and vegetables, and less minimally processed foods, moving away from the DietMed dietary pattern. Food shopping in specialised outlets can positively influence children’s dietary intake and the quality of their diet. Therefore, they would be more adherent to healthy dietary patterns, with higher consumption of fruits and vegetables, and minimally processed foods. This will have a positive impact on children’s health and preventing future chronic diseases, such as type 2 diabetes mellitus, obesity, and cardiovascular disease. Improving nutrition from infancy is not only essential to ensure optimal physical and cognitive development, but also contributes to reduce the incidence of nutritional deficiencies and chronic diseases in long term, thus alleviating the burden on public health systems. To achieve these benefits, it is essential to implement measures such as nutrition educational programmes in schools and communities, regulations and subsidies that make healthy foods more accessible, clear labelling policies that improve food purchasing decisions, and improving access to specialised shops in underserved areas. With these actions, a healthier food environment can be created to benefit both children and society as a whole and can lay the foundation for a healthy lifestyle throughout life.

## Figures and Tables

**Table 1:** Sociodemographic characteristics of children and parents according to food purchasing establishment.

	Hypermarket ^§^ (*n* = 315)	Supermarket ^§^ (*n* = 632)	Specialised Trade ^§^ (*n* = 201)	*p*-Value ^‡^
	Mean (SD)	Mean (SD)	Mean (SD)	
Kid Age (years) *	5.03 (0.89)	4.94 (0.80)	5.10 (1.10)	0.045
Kid BMI (kg/m^2^)	15.79 (1.75)	15.71 (1.74)	15.52 (1.69)	0.287
Zscore Kid BMI	0.13 (1.12)	0.07 (1.16)	−0.06 (1.19)	0.201
Waist circumference (cm)	53.37 (5.52)	53.24 (5.44)	53.21 (4.90)	0.931
Hip circumference (cm)	58.60 (6.58)	58.08 (6.67)	58.38 (5.56)	0.542
Father’s age (years) *	40.61 (6.17)	40.56 (5.03)	40.26 (6.52)	0.795
Mother’s age (years) *	39.19 (4.31)	39.16 (4.28)	40.33 (4.19)	0.005
	*n* (%)	*n* (%)	*n* (%)	
Kids’ sex				0.256
Male Female	155 (49.2) 160 (50.8)	312 (49.4) 320 (50.6)	112 (55.7) 89 (44.3)	
Kids’ weight status				0.469
Underweight	35 (11.1)	88 (13.9)	29 (14.4)	
Healthy weight	198 (62.9)	389 (61.6)	134 (66.7)	
Overweight	22 (7.0)	55 (8.7)	13 (6.5)	
Obesity	14 (4.4)	20 (3.2)	4 (2.0)	
Father’s educational level: No studies Primary Education Vocational Education and Training University degree Master’s or doctorate	3 (1.0) 32 (10.2) 70 (22.2) 103 (32.7) 59 (18.7)	10 (1.6) 67 (10.6) 168 (26.6) 228 (36.1) 76 (12.0)	2 (1.0) 20 (10.0) 42 (20.9) 67 (33.3) 47 (23.4)	0.016
Mother’s educational level: No studies Primary Education Vocational Education and Training University degree Master’s or doctorate	0 (0.0) 10 (3.2) 46 (14.6) 142 (45.1) 71 (22.5)	2 (0.3) 22 (3.5) 91 (14.4) 303 (47.9) 133 (21.0)	0 (0.0) 2 (1.0) 33 (16.4) 100 (49.8) 45 (22.4)	0.659

Abbreviations: BMI: Body Mass Index; SD: Standard Deviation. ^§^ Grouping variable = depending on the answer to the question “in which type of establishment do you shop?” * Ages refer to the time of answering the baseline questionnaire. ^‡^ Differences in prevalence across groups were examined using χ^2^ and ANOVA.

**Table 2:** Factors influencing the choice of establishment and foods according to food purchasing establishment.

	Hypermarket ^§^ (*n* = 315)	Supermarket ^§^ (*n* = 632)	Specialised Trade ^§^ (*n* = 201)	*p*-Value ^‡^
	*n* (%)	*n* (%)	*n* (%)	
Most influential factor in establishment choice: Distance Prices Quality of products Variety of product offerings Others	49 (15.6) 51 (16.2) 139 (44.1) 68 (21.6) 8 (2.5)	148 (23.4) 84 (13.3) 255 (40.3) 130 (20.6) 14 (2.2)	13 (6.5) 2 (1.0) 155 (77.1) 18 (9.0) 13 (6.5)	<0.001
Most influential factor in food choice: Organic Sustainable Brand Price Nutritional composition Product presentation Others	20 (6.3) 5 (1.6) 37 (11.7) 70 (22.2) 147 (46.7) 14 (4.4) 21 (6.7)	35 (5.5) 28 (4.4) 44 (7.0) 150 (23.7) 314 (49.7) 29 (4.6) 29 (4.6)	37 (18.4) 12 (6.0) 7 (3.5) 16 (8.0) 101 (50.2) 9 (4.5) 15 (7.5)	<0.001

^§^ Grouping variable, depending on the answer to the question “in which type of establishment do you shop?” ^‡^ Differences in prevalence across groups were examined using χ^2^.

**Table 3:** Food intake of according to food purchasing establishment.

		Hypermarket ^§^ (*n* = 315)	Supermarket ^§^ (*n* = 632)	Specialised Trade ^§^ (*n* = 201)	*p*-Value ^‡^
Milk and dairy (g/d)		408.47 (265.31)	398.45 (284.38)	414.52 (266.09)	0.517
Eggs (g/d)		17.55 (9.91)	18.85 (11.47)	19.00 (11.79)	0.315
White meat (g/d)		35.59 (18.97)	38.28 (27.45)	36.29 (19.63)	0.529
Red meat (g/d)		39.23 (30.27)	36.03 (27.70)	36.19 (24.74)	0.124
Processed meat (g/d)		61.25 (32.34) ^b^	57.77 (31.36)	57.34 (32.80) ^b^	0.044
Processed Fish (g/d)		6.27 (8.92)	6.63 (8.39)	5.47 (6.74)	0.975
Seafood (g/d)		2.52 (2.71)	2.64 (3.16)	2.80 (3.07)	0.980
Bluefish (g/d)		10.06 (9.94)	10.02 (10.24)	12.36 (11.06)	0.169
Whitefish (g/d)		18.23 (11.56)	16.34 (10.68)	18.45 (11.02)	0.083
Vegetables (g/d)		231.43 (179.48) ^b^	214.15 (146.27) ^c^	263.26 (174.23) ^b,c^	<0.001
Olive Oil (g/d)		18.12 (15.53) ^b^	16.99 (13.68) ^c^	20.86 (14.39) ^b,c^	0.006
Vegetable oil (g/d)		1.16 (3.86)	0.94 (2.99)	0.66 (1.84)	0.826
Other Fats (g/d)		5.49 (9.21)	5.05 (9.47)	4.05 (6.51)	0.477
Fruits (g/d)		329.01 (380.77) ^b^	337.94 (273.59)	359.45 (238.09) ^b^	0.002
Legumes (g/d)		32.55 (20.51) ^a^	29.19 (19.77) ^a^	31.42 (19.21)	0.014
Refined cereals (g/d)		70.87 (45.70)	71.96 (41.94)	69.66 (41.47)	0.600
Whole grains (g/d)		10.53 (20.44)	9.44 (17.09)	12.98 (22.84)	0.478
Potatoes (g/d)		25.44 (20.03)	27.97 (23.16)	31.21 (29.21)	0.108
Snacks (g/d)		11.03 (10.93)	10.43 (10.32)	10.01 (8.93)	0.590
Sugary beverages (ml/d)		160.04 (177.95)	128.40 (140.83)	144.05 (154.28)	0.075
Artificially sweetened beverages (ml/d)		3.23 (10.27)	3.02 (10.62)	1.42 (4.82)	0.414
Energy drinks (ml/d)		0.77 (10.08)	0.07 (1.25)	0.00 (0.00)	-
Sweets and pastries (g/d)		95.03 (68.76) ^a,b^	85.26 (68.64) ^a^	80.67 (57.34) ^b^	0.013
Convenience foods (g/d)		31.64 (21.13)	29.64 (18.94)	28.12 (27.38)	0.028
Nuts (g/d)		5.77 (9.38)	5.22 (7.60)	6.57 (10.85)	0.256

Values are mean and Standard Deviation (SD). Standard Deviation. ^§^ Grouping variable = depending on the answer to the question “in which type of establishment do you shop?” ^‡^ Differences in means between groups were tested by one-way ANOVA and Bonferroni’s post-hoc and were tested by UNIANOVA adjusted by kid’s and mother’s age, and father’s educational level. Different letters in rows shows statistically significant differences between groups (^a,b,c^) by the Bonferroni’s post-hoc test (*p* < 0.05).

**Table 4:** Diet quality (g/d) of according to food purchasing establishment.

		Hypermarket ^§^ (*n* = 315)	Supermarket ^§^ (*n* = 632)	Specialised Trade ^§^ (*n* = 201)	*p*-Value ^‡^
NOVA groups					
Group 1: Unprocessed or minimally processed foods		1127.72 (605.52)	1096.05 (531.68) ^c^	1183.55 (484.81) ^c^	0.010
Group 2: Oils, fats, salt and sugar		27.07 (21.61)	25.22 (18.76)	28.08 (17.46)	0.123
Group 3: Processed foods		63.66 (47.00)	62.41 (39.67)	67.15 (45.53)	0.506
Group 4: Ultra-processed foods		428.10 (253.97)	391.23 (217.83)	404.26 (231.53)	0.078

Values are mean and Standard Deviation (SD). ^§^ Grouping variable = depending on the answer to the question “in which type of establishment do you shop?” ^‡^ Differences in means between groups were tested by one-way ANOVA and Bonferroni’s post-hoc and were tested by UNIANOVA adjusted by kid’s and mother’s age, and father’s educational level. Different letters in rows shows statistically significant differences between groups (^a,b,c^) by the Bonferroni’s post-hoc test (*p* < 0.05).

**Table 5:** Nutrient intake according to food purchasing establishment.

	Hypermarket ^§^ (*n* = 315)	Supermarket ^§^ (*n* = 632)	Specialised Trade ^§^ (*n* = 201)	*p*-Value ^‡^
Energy (Kcal)	2158.97 (609.67)	2041.62 (545.33)	2118.55 (498.69)	0.154
Carbohydrates (g/day)	233.55 (78.32)	222.98 (65.99)	230.35 (56.33)	0.262
Proteins (g/day)	91.31 (22.85)	87.06 (25.08)	88.54 (21.94)	0.228
Lipids (g/day)	95.50 (31.64)	89.05 (27.95)	93.66 (28.16)	0.132
MUFAs (g/day)	36.96 (15.46)	34.36 (12.86)	37.35 (13.04)	0.135
PUFAs (g/day)	11.35 (4.93)	10.60 (4.08)	10.81 (3.61)	0.299
SFA (g/day)	27.04 (9.44) ^a^	25.04 (8.51) ^a^	25.55 (9.27)	0.039
w-6 FA (g/day)	352.52 (137.49)	342.84 (134.14)	343.86 (127.57)	0.374
w-3 FA (g/day)	288.32 (120.23)	275.37 (104.90)	287.43 (97.77)	0.192
Cholesterol (mg/day)	286.40 (85.35)	277.72 (86.06)	275.22 (82.58)	0.388
Folate (μg/day)	321.74 (124.48)	308.14 (114.22) ^c^	337.83 (112.22) ^c^	0.007
Fiber (g/day)	22.19 (9.23) ^b^	21.47 (7.80) ^c^	23.60 (7.36) ^b,c^	0.002
Vitamin A (µg/day)	1205.48 (678.70)	1115.58 (594.04)	1199.95 (538.44)	0.255
Vitamin B_1_ (mg/day)	1.52 (0.46)	1.45 (0.42)	1.53 (0.37)	0.149
Vitamin B_2_ (mg/day)	2.14 (0.73)	2.06 (0.73)	2.23 (0.82)	0.268
Vitamin B_3_ (mg/day)	37.63 (11.50)	36.32 (11.29)	38.44 (10.46)	0.282
Vitamin B_6_ (mg/day)	2.44 (0.79)	2.35 (0.73) ^c^	2.53 (0.62) ^c^	0.008
Vitamin B_12_ (µg/day)	5.05 (2.03)	4.77 (1.76)	4.80 (1.55)	0.337
Vitamin C (mg/day)	153.38 (98.42)	148.29 (91.55) ^c^	161.29 (67.09) ^c^	0.003
Vitamin D (µg/day)	3.37 (2.26)	3.26 (2.18)	3.68 (2.31)	0.120
Vitamin E (mg/day)	9.18 (4.70)	8.62 (4.15) ^c^	9.41 (4.23) ^c^	0.035
Phosphor (mg/day)	1901.70 (1037.42)	1752.17 (891.08)	1840.22 (840.48)	0.229
Magnesium (mg/day)	319.07 (98.68) ^a^	303.10 (85.04) ^a,c^	325.32 (74.88) ^c^	0.011
Iron (mg/day)	14.76 (4.44)	14.10 (3.83)	14.75 (3.27)	0.153
Iodine (µg/day)	113.82 (35.72)	110.18 (34.26)	115.70 (37.08)	0.727
Potassium (mg/day)	3664.03 (1410.18)	3472.51 (1130.04) ^c^	3740.19 (950.50) ^c^	0.010
Calcium (mg/day)	1259.72 (433.45)	1186.05 (428.62)	1235.21 (423.11)	0.159
Corm (µg/day)	67.76 (36.40)	63.25 (32.13) ^c^	72.07 (36.54) ^c^	0.004
Sodium (mg/day)	3041.59 (1139.00)	2954.83 (1145.96)	3106.02 (1154.85)	0.896
Selenium (µg/day)	74.83 (22.59)	72.10 (19.68)	74.33 (18.41)	0.552
Zinc (mg/day)	10.17 (3.27)	9.73 (3.25)	10.42 (3.74)	0.216

Values are mean and Standard Deviation (SD). ^§^ Grouping variable = depending on the answer to the question “in which type of establishment do you shop?” ^‡^ Differences in means between groups were tested by one-way ANOVA and Bonferroni’s post-hoc and were tested by UNIANOVA adjusted by kid’s and mother’s age, and father’s educational level. Different letters in rows shows statistically significant differences between groups (^a,b,c^) by the Bonferroni’s post-hoc test (*p* < 0.05).

**Table 6:** Association between food purchasing establishment and diet quality.

		Specialised Trade (*n* = 201)	Hypermarket (*n* = 315)	Supermarket (*n* = 632)
Q1. Skips breakfast	Crude OR	1.00 (ref.)	1.46 (0.62–3.43)	0.95 (0.42–2.16)
	Adjusted OR	1.00 (ref.)	0.72 (0.28–1.84)	1.12 (0.46–2.73)
Q2. Takes dairy product for breakfast	Crude OR	1.00 (ref.)	1.23 (0.77–1.96)	1.33 (0.87–2.02)
	Adjusted OR	1.00 (ref.)	1.20 (0.72–2.00)	1.27 (0.81–2.00)
Q3. Takes cereal or grains product for breakfast	Crude OR	1.00 (ref.)	0.54 (0.36–0.80) **	0.63 (0.43–0.90) *
	Adjusted OR	1.00 (ref.)	0.51 (0.33–0.79) **	0.59 (0.40–0.89) **
Q4. Takes pastries/commercially baked goods for breakfast	Crude OR	1.00 (ref.)	1.38 (0.87–2.18)	1.39 (0.93–2.09)
	Adjusted OR	1.00 (ref.)	0.75 (0.46–1.22)	0.63 (0.41–0.97) *
Q5. Takes a fruit or fruit juice daily	Crude OR	1.00 (ref.)	0.71 (0.37–1.35)	0.78 (0.43–1.41)
	Adjusted OR	1.00 (ref.)	0.85 (0.33–2.22)	0.88 (0.37–2.09)
Q6. Takes a second serving of fruit daily	Crude OR	1.00 (ref.)	0.63 (0.41–0.97) *	0.61 (0.41–0.90) *
	Adjusted OR	1.00 (ref.)	0.61 (0.37–1.01)	0.58 (0.37–0.92) *
Q7. Consumes yogurts and/or 40 g cheese daily	Crude OR	1.00 (ref.)	0.76 (0.44–1.32)	0.64 (0.39–1.05)
	Adjusted OR	1.00 (ref.)	0.79 (0.44–1.43)	0.70 (0.41–1.18)
Q8. Consumes raw or cooked vegetables daily	Crude OR	1.00 (ref.)	0.48 (0.28–0.82) **	0.44 (0.27–0.73) **
	Adjusted OR	1.00 (ref.)	0.36 (0.18–0.73) **	0.33 (0.17–0.63) **
Q9. Consumes raw or cooked vegetables more than 1/day	Crude OR	1.00 (ref.)	0.63 (0.44–0.90) *	0.59 (0.42–0.81) **
	Adjusted OR	1.00 (ref.)	0.64 (0.43–0.95) *	0.59 (0.42–0.84) **
Q10. Regular fish consumption (at least 2–3/week)	Crude OR	1.00 (ref.)	0.78 (0.55–1.12)	0.60 (0.44–0.83) **
	Adjusted OR	1.00 (ref.)	0.81 (0.55–1.20)	0.63 (0.45–0.90) **
Q11. Goes >1/week fast food restaurant	Crude OR	1.00 (ref.)	1.48 (0.94–2.32)	1.54 (1.02–2.32) *
	Adjusted OR	1.00 (ref.)	1.31 (0.34–4.99)	1.90 (0.55–6.63)
Q12. Regular nut consumption (at least 2–3/week)	Crude OR	1.00 (ref.)	0.83 (0.55–1.25)	0.72 (0.50–1.04)
	Adjusted OR	1.00 (ref.)	0.85 (0.54–1.33)	0.76 (0.51–1.13)
Q13. Likes pulses and eats more than 1/week	Crude OR	1.00 (ref.)	1.05 (0.64–1.73)	0.66 (0.43–1.01)
	Adjusted OR	1.00 (ref.)	1.44 (0.77–2.69)	0.74 (0.44–1.23)
Q14. Takes sweets and candies several times every day	Crude OR	1.00 (ref.)	2.19 (0.59–8.08)	1.61 (0.46–5.64)
	Adjusted OR	1.00 (ref.)	0.63 (0.16–2.50)	0.60 (0.17–2.12)
Q15. Consumes rice or pasta almost daily (≥5 times per week)	Crude OR	1.00 (ref.)	1.01 (0.68–1.51)	0.91 (0.64–1.31)
	Adjusted OR	1.00 (ref.)	0.95 (0.62–1.47)	0.89 (0.61–1.31)
Q16. Use of olive oil at home	Crude OR	1.00 (ref.)	0.48 (0.13–1.74)	0.52 (0.15–1.78)
	Adjusted OR	1.00 (ref.)	0.54 (0.14–2.07)	0.61 (0.17–2.11)
Low MedDiet Adherence	Crude OR	1.00 (ref.)	1.62 (0.89–2.94)	1.89 (1.10–3.25) *
	Adjusted OR	1.00 (ref.)	1.63 (0.72–3.65)	2.12 (1.02–4.38) *
Medium MedDiet Adherence	Crude OR	1.00 (ref.)	1.28 (0.90–1.83)	1.09 (0.79–1.51)
	Adjusted OR	1.00 (ref.)	1.42 (0.96–2.10)	1.20 (0.85–1.69)
High MedDiet Adherence	Crude OR	1.00 (ref.)	0.61 (0.42–0.89) *	0.67 (0.48–0.94) *
	Adjusted OR	1.00 (ref.)	0.59 (0.39–0.90) *	0.65 (0.45–0.93) *

Values are Odds Ratio (95%CI). Abbreviations: CI: Confidence Interval. g/d: grams per day. ml/d: millilitres per day. METs: Metabolic Equivalent of Tasks. OR: Odds Ratio. § Grouping variable = depending on the answer to the question “in which type of establishment do you shop?” OR: Adjusted by kid’s and mother’s age and father’s educational level. * *p*-value < 0.05 and ** *p*-value < 0.01.

**Table 7:** Association between factors influencing the choice of establishment and foods and the type of food purchasing establishment.

		Specialised Trade (*n* = 201) OR (95% CI)	Hypermarket (*n* = 315) OR (95% CI)	Supermarket (*n* = 632) OR (95% CI)
Most influential factor in the establishment decision:				
Distance	Crude OR	1.00 (ref.)	2.66 (1.40–5.05) **	4.43 (2.45–8.00) **
	Adjusted OR	1.00 (ref.)	2.46 (1.25–4.83) **	3.99 (2.15–7.42) **
Prices	Crude OR	1.00 (ref.)	19.22 (4.62–79.90) **	15.28 (3.72–62.69) **
	Adjusted OR	1.00 (ref.)	18.30 (4.38–76.48) **	13.99 (3.39–57.62) **
Quality of products	Crude OR	1.00 (ref.)	0.23 (0.16–0.35) **	0.20 (0.14–0.29) **
	Adjusted OR	1.00 (ref.)	0.24 (0.15–0.36) **	0.20 (0.14–0.30) **
Variety of product offerings	Crude OR	1.00 (ref.)	2.80 (1.61–4.87) **	2.64 (1.57–4.44) **
	Adjusted OR	1.00 (ref.)	2.70 (1.49–4.90) **	2.79 (1.60–4.86) **
Others	Crude OR	1.00 (ref.)	0.38 (0.15–0.92) *	0.33 (0.15–0.71) **
	Adjusted OR	1.00 (ref.)	0.44 (0.18–1.12)	0.34 (0.15–0.76) **
Most influential factor in food choices:				
Organic	Crude OR	1.00 (ref.)	0.29 (0.16–0.52) **	0.25 (0.15–0.41) **
	Adjusted OR	1.00 (ref.)	0.29 (0.15–0.56) **	0.23 (0.13–0.41) **
Sustainable	Crude OR	1.00 (ref.)	0.25 (0.09–0.72) *	0.71 (0.36–1.43)
	Adjusted OR	1.00 (ref.)	0.30 (0.90–1.00)	0.83 (0.37–1.85)
Brand	Crude OR	1.00 (ref.)	3.62 (1.58–8.30) **	2.03 (0.90–4.59)
	Adjusted OR	1.00 (ref.)	3.70 (1.50–9.14) **	2.34 (0.97–5.63)
Price	Crude OR	1.00 (ref.)	3.24 (1.82–5.77) **	3.53 (2.05–6.07) **
	Adjusted OR	1.00 (ref.)	2.69 (1.48–4.89) **	2.92 (1.68–5.10) **
Nutritional composition	Crude OR	1.00 (ref.)	0.84 (0.58–1.19)	0.94 (0.68–1.30)
	Adjusted OR	1.00 (ref.)	0.84 (0.57–1.24)	0.97 (0.69–1.37)
Product presentation	Crude OR	1.00 (ref.)	0.97 (0.41–2.30)	1.00 (0.47–2.16)
	Adjusted OR	1.00 (ref.)	0.98 (0.41–2.36)	0.93 (0.42–2.03)
Others	Crude OR	1.00 (ref.)	0.87 (0.44–1.73)	0.58 (0.31–1.11)
	Adjusted OR	1.00 (ref.)	0.86 (0.41–1.78)	0.53 (0.27–1.06)

Values are Odds Ratio (95%CI). Abbreviations: CI: Confidence Interval. OR: Odds Ratio. ^§^ Grouping variable = depending on the answer to the question “in which type of establishment do you shop?” OR: Adjusted by kid’s and mother’s age, father’s educational level. * *p*-value < 0.05 and ** *p*-value < 0.01.

## Data Availability

There are restrictions on the availability of data for this trial, due to the signed consent agreements around data sharing, which only allow access to external researchers for studies following the project purposes. Requestors wishing to access the trial data used in this study can make a request to pep.tur@uib.es.

## Supplementary Materials

The following supporting information can be downloaded at: https://www.mdpi.com/article/10.3390/foods13182930/s1, Table S1: Classification according to NOVA System.

## Abbreviations

FFQ: Food Frequency Questionnaire; HPM: hypermarkets; MedDiet: Mediterranean Diet; SES: specialized establishments; SPM: supermarkets; UPS: Ultra-Processed Foods.
