# Supplementary material for: Preschool Children’s Dietary Patterns Are Associated with Food Shopping Establishments: The SENDO Project
Source: Foods. 2024 Sep 16;13(18):2930. doi: 10.3390/foods13182930 (PMC11431715; doi:10.3390/foods13182930)
Supplement: Supplementary file 1 [file foods-13-02930-s001.zip › foods-3143904-supplementary.pdf]

[1] **Supplementary Table S1.** Classification of food according to the NOVA System.

Group 1: Unprocessed or minimally processed foods	Apple, asparagus, eggplant, avocado, banana, beans, cabbage, carrot, chard, cherry, chicken, clam, curd, eggs, fig, fish, fruit juice, fruit smoothie, garbanzo beans, grapes, kiwi fruit, lamb, leek, lentils, lettuce, mango, meatball, melon, milk (skimmed or whole), nuts, octopus, onion, orange, pasta, peas, peach, pear, pepper, pineapple, plum, pork, potatoes, seafood, pumpkin, rabbit meat, rice, strawberry, string beans, tangerine, tomato, veal, viscera, watermelon.
Group 2: Processed culinary ingredients	Sunflower oil, olive oil, sugar, butter, cream, salt.
Group 3: Processed foods	Olives, compote of fruit, cured ham, canned fish, jam, baguette, wholemeal bread, white cheese, cured cheese, bacon.
Group 4: Ultra-processed food and drink products	Bakery products, blood sausage, bonbon, breakfast cereals, cake, candies, carbonated beverages, cereal bar, chocolate bar, chocolate powder, cookies, crab sticks, cream cheese, cream chocolate, croquet*, cruller or 'churro'*, custard*, dry soup, fish sticks, gelatine, ham, hamburger, ice cream, industrialised juices (sugar-sweetened juices), industrialised sliced cheese, industrialised sliced bread, ketchup, lasagne*, margarine, mayonnaise*, muffin*, nougat, nugget, pâté, petit suisse, pie*, pizza*, popcorn*, salami, sausage, pepperoni, snacks, soda, soft drinks, sweetened beverages, sweetened fermented milk, sweetened yogurt (skimmed or whole).

[1]The classification of each item of the Food Frequency Questionnaire (FFQ) used in the SENDO and into one of the four food groups according to NOVA system.

* There are foods that could have different ratings depending on the way they are prepared: homemade or industrialised. In these cases, we chose to classify them as ultra-processed foods because industrial food products in supermarkets have replaced most traditional foods.
